# Real-Time Correction and Stabilization of Laser Diode Wavelength in Miniature Homodyne Interferometer for Long-Stroke Micro/Nano Positioning Stage Metrology

**DOI:** 10.3390/s19204587

**Published:** 2019-10-22

**Authors:** Yindi Cai, Baokai Feng, Qi Sang, Kuang-Chao Fan

**Affiliations:** Key Laboratory for Micro/Nano Technology and System of Liaoning Province, Dalian University of Technology, Dalian 116024, China; fengbaokai@mail.dlut.edu.cn (B.F.); fan@dlut.edu.cn (K.-C.F.)

**Keywords:** homodyne interferometer, laser diode, wavelength correction, wavelength stabilization, positioning stage

## Abstract

A low-cost miniature homodyne interferometer (MHI) with self-wavelength correction and self-wavelength stabilization is proposed for long-stroke micro/nano positioning stage metrology. In this interferometer, the displacement measurement is based on the analysis of homodyne interferometer fringe pattern. In order to miniaturize the interferometer size, a low-cost and small-sized laser diode is adopted as the laser source. The accuracy of the laser diode wavelength is real-time corrected by the proposed wavelength corrector using a modified wavelength calculation equation. The variation of the laser diode wavelength is suppressed by a real-time wavelength stabilizer, which is based on the principle of laser beam drift compensation and the principle of automatic temperature control. The optical configuration of the proposed MHI is proposed. The methods of displacement measurement, wavelength correction, and wavelength stabilization are depicted in detail. A laboratory-built prototype of the MHI is constructed, and experiments are carried out to demonstrate the feasibility of the proposed wavelength correction and stabilization methods.

## 1. Introduction

Long-stroke micro/nano positioning stages with high accuracy act as fundamental units in various fields, such as precision machining and precision measurement [1,2]. A high-precision position sensor is required to be integrated into the positioning stages for closed-loop feedback control of the axis’s motion. Thus, the measurement accuracy of the position sensor is one of the key factors that affects the positioning accuracy of the stage.

For advantages of fast and long-stroke measurement with nanometer accuracy, commercial laser interferometers (CLIs) are widely used as position feedback sensors of a stage in precision machining or measurement equipment, such as mask aligners, wafer steppers, and ultra-precision measuring machines. A frequency/wavelength stabilized He-Ne laser is generally adopted as the laser source of the CLI, resulting in the whole system being rather bulky and expensive [1,3]. However, for small-sized micro/nano positioning stages commonly used in Micro Electro Mechanical Systems (MEMS) equipment or micro/nano machining, those CLIs would not be able to integrate into the systems as a position sensor. To address the problem of bulky size and high-cost, many interferometers with a laser diode as the laser source were developed [4,5,6,7,8,9,10]. Some of them have been embedded into micro/nano positioning stages as a position sensor [4,5,6,7]. In such an interferometer, the displacement information is often encoded by the phase-to-intensity change of the interference signals. The wavelength of the laser source is often treated as the length unit of those interferometers. However, the laser diode suffers from the short temporal coherence length and low stability in output power and wavelength. Therefore, the necessary condition to use laser diode as a laser source in an interferometer is to stabilize and correct its wavelength.

There are a huge number of methods and techniques for stabilizing laser diode wavelength. Generally, it can be divided into two categories, namely active stabilization and passive stabilization [8,11,12,13,14,15,16,17]. Active stabilization methods usually involve certain kinds of electronic feedback systems in which variations of some parameters are converted to an electronic signal to stabilize the laser wavelength [13]. The stability of the wavelength with such active stabilization system can be achieved to 10^−9^ [14,15]. However, due to complexity and the high-cost of realization, active stabilization methods cannot be applied in a small-sized interferometer. Since the laser diode frequency/wavelength is sensitive to the temperature and supplied current, it is important to apply a current source and temperature controller to stabilize the laser frequency/wavelength, which is called the passive stabilization methods [8,11,12,13,14,15,16,17]. The passive stabilization methods are usually simple and easy implement. In addition, since the laser diode temperature is maintained in a specific range, laser diode mode hopping can be avoided [8].

In addition, wavelength is the length unit of the laser interferometers. Therefore, it is essential to ensure the accuracy of the laser diode wavelength in the displacement measurement. The laser wavelength is conventionally measured by the methods based on the Edlen equation [18] or its modified equations [19,20], interference [21,22], optical beating [23,24], optical frequency combs [25,26,27], and specific wavelength-dependent material properties [5,6,7,28,29]. The laser wavelength can be compensated by combining the Edlen equation or its modified equation with an air sensor. This method is commonly used in the commercial laser interferometers [30,31]. However, those equations were obtained based on the properties of the He-Ne laser, which is not compatible with the laser diode. In addition, the measurement accuracy is significantly dependent on the sensitivity and accuracy of the air sensor. In the methods based on the principles of interference and optical beating, a high-precision reference laser is necessary, and the measurement accuracy depends on the reference laser. Moreover although those methods have a high accuracy, they are complicated in system construction and are high-cost, and so they are seldom used in industry. The methods based on optical frequency combs can achieve a high measurement accuracy. However, the femtosecond laser has a bulky size and high-cost. Among those methods, the simplest and most applicable one is the method based on the specific material properties. In our previous research, a laser diode interferometer (LDI) was designed for the displacement feedback of a nanopositioning stage [7] in which a low-cost and small-sized laser diode was adopted as the light source. A wavelength corrector, consisting of a grating and an autocollimator, was designed for correcting the wavelength in the LDI based on the diffraction grating equation. Since only the variation of diffraction angle was considered, the measurement accuracy of the laser diode wavelength was low. Moreover, a plane mirror was applied as the optical reflector of the MLDI [7]. The tilt motion errors of the plane mirror would affect the measurement range and accuracy [28].

Therefore, in this paper, a novel miniature homodyne interferometer (MHI) is designed based on the previous works [6,7]. In this MHI, a modified wavelength calculation equation, is proposed, from which a real-time wavelength corrector (RWC) is designed. It is verified that the wavelength measurement accuracy of the newly proposed RWC is better than that in Ref. [7]. In addition, in order to improve the repeatability of the proposed MHI, a real-time wavelength stabilizer (RWS) based on the laser beam drift compensation principle and automatic temperature control principle is presented to stabilize the laser diode wavelength. Moreover, the plan mirrors in Ref. [6,7] is replaced by two corner cube retroreflectors in the proposed MHI, by which the measurement range is enlarged. This article demonstrates the novelty of the design and construction of the prototype system. A series of experiments are preformed to verify the feasibility of the proposed MHI, RWC, and RWS.

## 2. Optical Configuration

**Figure 1:**
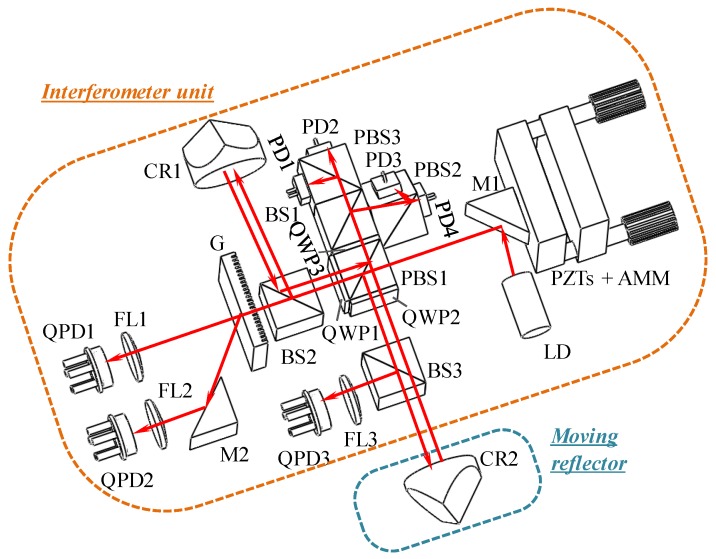
Optical configuration for the miniature homodyne interferometer (MHI).

Figure 1 shows the optical configuration of the miniature homodyne interferometer (MHI) with self-wavelength correction and self-wavelength stabilization. The MHI includes a stationary interferometer unit and a moving reflector. There are three sets in the interferometer unit. Set I is a Michelson interferometer-based laser diode interferometer (LDI), which is composed of three polarizing beam splitters (PBS, PBS2, PBS3), two beam splitters (BS1, BS2), three quarter waveplates (QWP1, QWP2, QWP3), four photodetectors (PD1, PD2, PD3, PD4), and a corner cube retroreflector (CR1). Set II is a real-time wavelength corrector (RWC), which consists of a diffraction grating (G), two focus lenses (FL1, FL2), two quadrant-photodetectors (QPD1, QPD2), and a PZT driven angle mirror mount (AMM). Set Ⅲ, consisting of two thermoelectric cools (TECs), a thermistor, and two heatsinks, is a real-time wavelength stabilizer (RWS). CR2 is employed as the moving reflector of the proposed MHI. 

## 3. Measurement Principle

**Figure 2:**
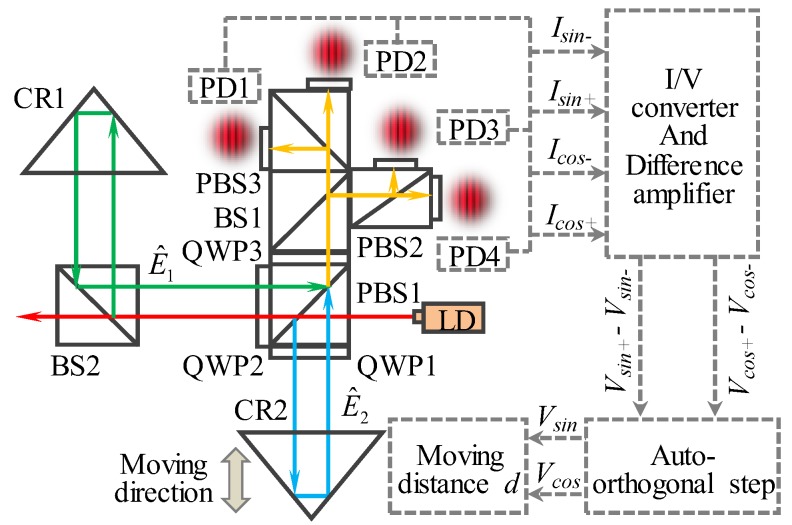
Principle and data processing procedure of the laser diode interferometer (LDI).

The principle and data processing procedure of the displacement measurement based on the homodyne interferometer fringe pattern analysis are shown in Figure 2. A linearly polarized beam emitted from a laser diode is split into a reference beam and a measurement beam by PBS1. The reference beam is reflected by CR1 and passes through QPW2 twice before going back to PBS1. The reflected measurement beam, which is reflected by CR2, passes through QPW1 twice before going back to PBS1, combining with the reflected reference beam. The combined beams (${\hat{E}}_{1},\;{\hat{E}}_{2}$) interfere with each other. The interference signals are then projected onto four detectors (PD1 to PD4) after phase controlled by two PBSs (PBS2, PBS3), a BS (BS1), and a QWP (QWP3). Therefore, the intensity of each detector can be expressed as follows:

(1) $$\left\{ \begin{array}{l} I_{PD1}=A^{2}[1+\sin(\Delta \varphi )]\\ I_{PD2}=A^{2}[1-\sin(\Delta \varphi )]\\ I_{PD3}=A^{2}[1+\cos(\Delta \varphi )]\\ I_{PD4}=A^{2}[1-\sin(\Delta \varphi )] \end{array}\right.,$$

where *A* represents the intensity magnitude of the combined beams. Δ*φ* is the phase difference of ${\hat{E}}_{1}$ and ${\hat{E}}_{2}$, which can be calculated by combining the sinusoidal and cosine signals. According to *I_PD1_* to *I_PD4_*, the sinusoidal and cosine signals can be obtained.

(2) $$\left\{ \begin{array}{l} I_{PD1}-I_{PD2}=A^{2}\sin(\Delta \varphi )\\ I_{PD3}-I_{PD4}=A^{2}\cos(\Delta \varphi ) \end{array}\right.,$$

The motion of CR2 would cause an optical path difference (2Δ*d*) between the two combined beams. Therefore, the moving distance *d* of CR2 with respect to the interferometer unit can be calculated by combining the integer and fraction fringe counts,

(3) $$\Delta d=\frac{\lambda }{2n}(N+\frac{\Delta \varphi_{0}+\Delta \varphi_{f}}{2\pi }),$$

where, *N* is the integer fringe count and $\frac{\Delta \varphi_{0}+\Delta \varphi_{f}}{2\pi }$ is the fraction fringe count. $\Delta \varphi_{0}$ and $\Delta \varphi_{f}$ represent the phases of initial and final incomplete wave cycles, respectively.

Although the laser diode can reduce the size and cost of the LDI, it suffers from the low stability in output power and wavelength. It is known that the laser wavelength is the length unit of the interferometry. The accuracy and the stability of the wavelength significantly influence the measurement accuracy of the interferometry. Therefore, in order to achieve a high precision measurement and improve the stability of the measurement system, it is essential to correct and stabilize the laser diode wavelength in real-time. 

**Figure 3:**
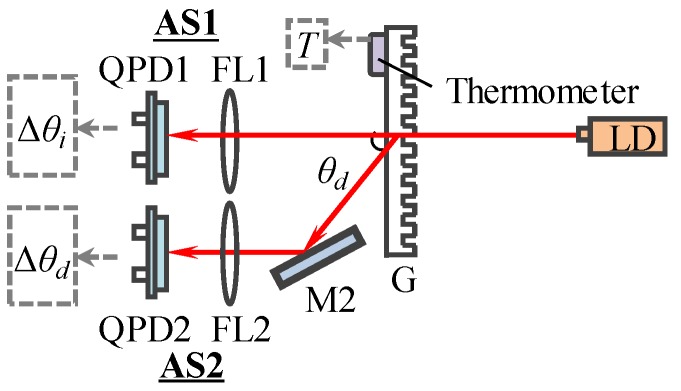
Principle of the real-time wavelength corrector (RWC).

Figure 3 shows the principle of correcting laser diode wavelength. In our previous research, the variation of the diffraction angle (Δ*θ_d_*) is the only variable in the wavelength calculation equation [7]. However, the laser diode has the property of beam drift, which will cause the variation of the incidence angle (Δ*θ_i_*). Additionally, the grating pitch (*d*) will be changed with the variation of the ambient temperature. If the laser beam was set to normally project onto the grating (i.e. *θ_i_* was equal to zero), the wavelength calculation equation should be modified to:

(4) $$\lambda =[1-\alpha \cdot (25-T)]\cdot d\cdot [\sin(\Delta \theta_{i})+\sin(\theta_{d}+\Delta \theta_{d})],$$

where, *α* is thermal expansion coefficient of the grating. *T* is the temperature of the grating during the experiment, which can be detected by a thermometer. Δ*θ_i_* and Δ*θ_d_* represent the variations of the incident angle and diffraction angle, which can be detected by two autocollimator sets (AS1, AS2), respectively, as shown in Figure 3. Both autocollimator sets are composed of a focus lens (FL1, FL2) and a quadrant-photodetector (QPD1, QPD2). By using Equation (4), the laser diode wavelength can be real-time corrected.

**Figure 4:**
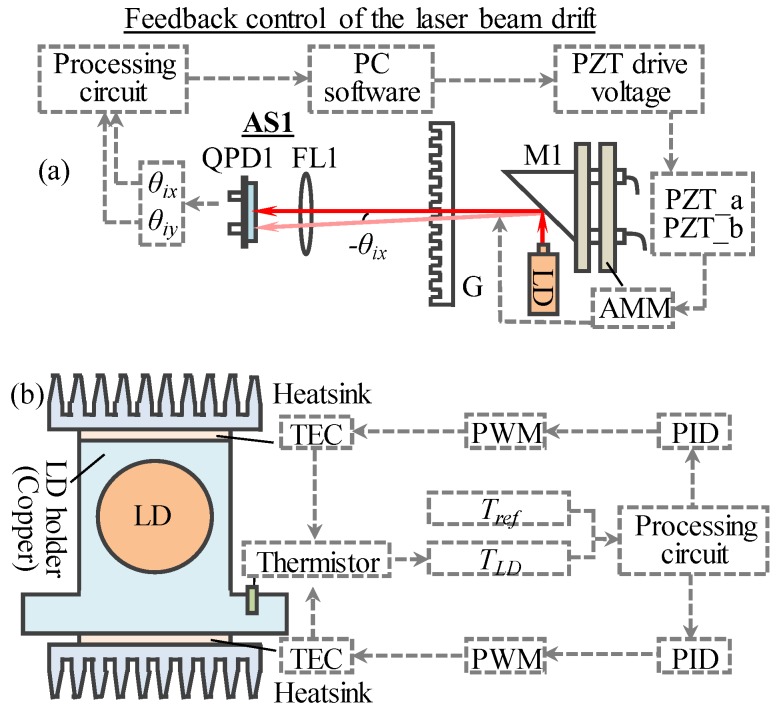
Principle of the real-time wavelength stabilizer: (**a**) method I compensation of laser beam drift, (**b**) method II automatic temperature control of laser diode.

The stability of the laser diode wavelength is another factor that effects the accuracy and repeatability of the LDI. Figure 4 shows two comprehensive wavelength stabilization methods. As show in Figure 4a, method I is based on the principle of the laser beam drift compensation, which has been introduced in our previous researches [32]. The output of AS1 is adopted to feedback control the angle of an angle mirror mount (AMM), in which two mini-PZTs (PZT_a, PZT_b) are embedded into the threaded shafts of the AMM. By rotating the AMM, the focused spot of the laser beam can remain at the center of QPD1. Method II is based on the principle of automatic temperature control (ATC), as shown in Figure 4b. A closed-loop control system is adopted to improve the precision of temperature control. A negative temperature coefficient (NTC) thermistor is installed in a copper holder of the laser diode to detect the laser diode’s surface temperature (*T_LD_*). After comparing with the reference temperature (*T_ref_*), a differential signal is generated and input to the PID controller. Then, two thermoelectric cools (TECs), which are fed by an external current generator, are regulated to heat or cool according to the differential signal. By using this closed-loop temperature control system, *T_LD_* can be effectively controlled within the commanded accuracy. Combined with method I and method II, the laser diode wavelength can be real-time stabilized.

In addition, the mode hopping should normally occur between adjacent modes of the laser diode when temperature varies. Thus, the laser diode temperature should be maintained in a range in which no laser diode mode hopping occurs [8]. Therefore, the proposed real-time wavelength stabilizer not only stabilized the wavelength, but also avoided the laser diode mode hopping.

## 4. Experiments and Results

### 4.1. Performance of the Real-Time Wavelength Corrector (RWC)

**Figure 5:**
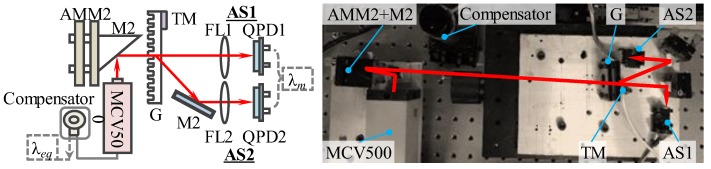
Experimental setup for investigating the performance of the RWC.

In order to verify the feasibility and accuracy of the proposed real-time wavelength corrector (RWC), an experiment setup was constructed, as shown in Figure 5. 

A wavelength stabilized He-Ne laser interferometer (MCV500, Optodyne, California, USA) with a wavelength of 632.694 nm, a laser stability of ±0.05 ppm, and an output power of 1.5 mW was adopted as the measured laser source. The wavelength of the He-Ne laser can be simultaneously calculated by the RWC and the wavelength compensator kit of the MCV500, which is based on the Edlen equation [17]. In the RWC, a 1200 line/ mm grating (Thorlab, Morganville, USA) was selected. Two high-precision QPDs (QPD1 and QPD2, QP5.8-6-TO5, First Sensor, Arne Wollmann, Germany) with a measurement resolution of 0.05 μm were applied to detect the variation of *θ_i_* and *θ_d_*, respectively.

**Figure 6:**
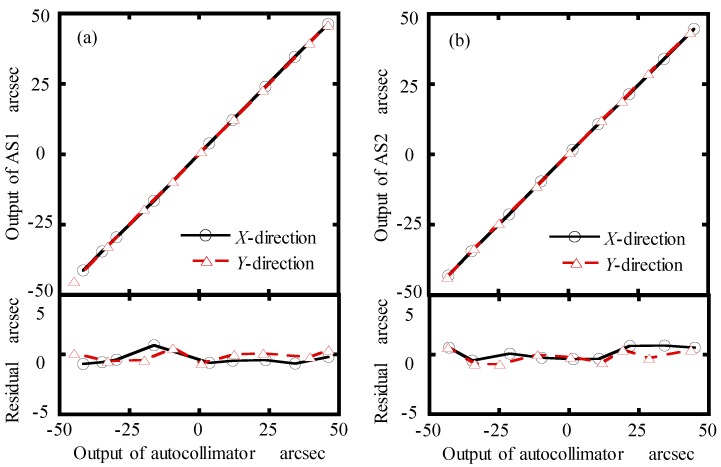
Calibration results of the AS1 and AS2 in the X- and Y-direction: (**a**) AS1, (**b**) AS2.

Prior to calculating the He-Ne laser wavelength, the AS1 and AS2 were calibrated by a commercial autocollimator (5000U3050, AutoMat, Tianjin, China), which has a measurement accuracy of 0.2 arcsec and repeatability of 0.05 arcsec. The calibration range was set to be ±50 arcsec. Figure 6 shows the calibration results of the AS1 and AS2 in the *X*- and *Y*-directions. It can be seen from the figure that the residual of each result was within ±0.4 arcsec.

**Figure 7:**
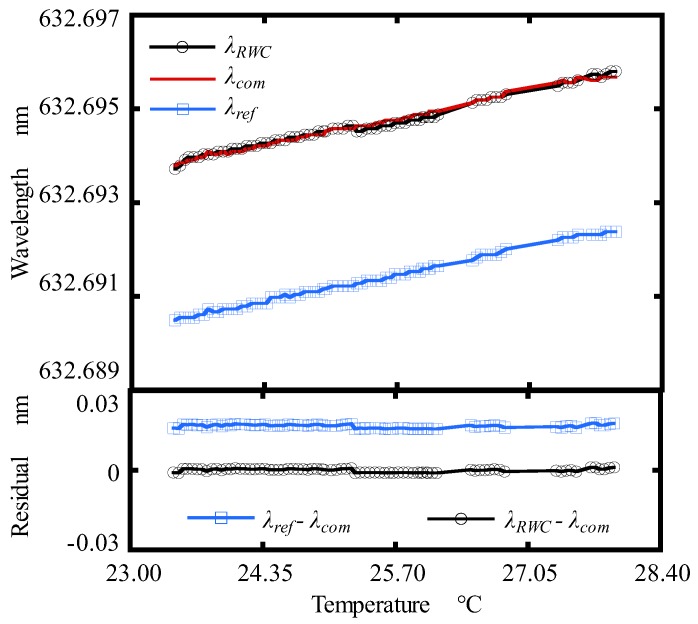
Calibration result of the RWC under various temperatures.

Then, the wavelength of the He-Ne laser was calculated by the RWC (*λ_RWC_*) and compensator (*λ_com_*) under the various temperatures. Figure 7 shows the variation of the laser diode wavelength with temperature. The missing measured points correspond to the temperature period when the MCV500 was not in operation. For comparison, the wavelength (*λ_ref_*) evaluated by the equation, which was proposed in Ref. [7], was also plotted in the figure. As seen from Figure 7, the He-Ne laser wavelength was enlarged with the increase of the temperature. The laser wavelength (*λ_RWC_*) obtained by the modified wavelength correction equation Equation (4) was larger than the wavelength (*λ_ref_*) obtained by Ref. [7]. *λ_RWC_* was in a good agreement with that calculated by compensator *λ_com_*. It indicates that the measurement accuracy of the wavelength corrector proposed in this research is much higher than that proposed in Ref. [7]. The maximum residual between *λ_RWC_* and *λ_com_* was evaluated to be ±0.007 nm, which is a satisfactory value for our designed long-stroke micro/nano positioning stage.

### 4.2. Performance of the Real-Time Wavelength Stabilizer (RWS)

**Figure 8:**
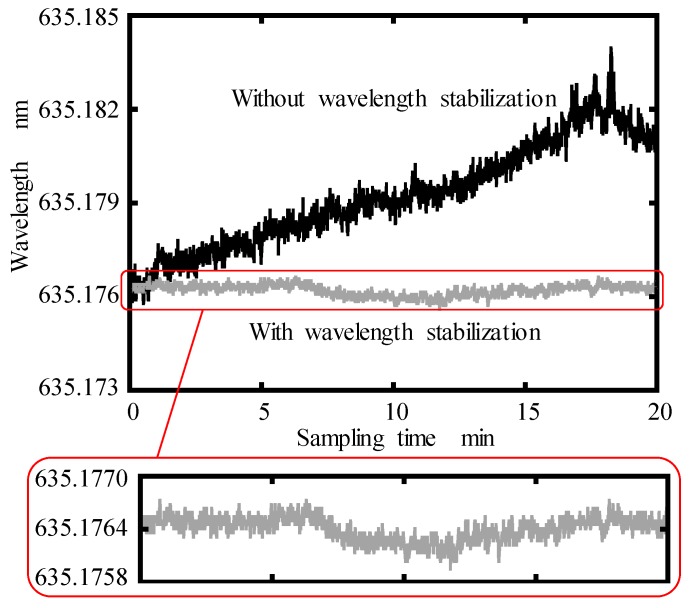
Real-time recording of the laser diode wavelength with and without stabilization.

The feasibility of the real-time wavelength stabilizer (RWS) was investigated by using a laser diode (DI635-2-3, Huanic, Xi’an, China), which has a nominal wavelength of 635 nm and an output power of 2 mW. The experiment results are shown in Figure 8.

The experimental setup for measuring the laser diode wavelength is similar to that shown in Figure 5, except the MCV500 was replaced by the studied laser diode. It has been confirmed that the AS1 and AS2 have the same angle measurement accuracy as indicated in Figure 6. In order to compensate the laser beam drift, an angle mirror mount integrated with two mini-PZTs was designed and constructed. In the ATC set, a negative temperature coefficient (NTC) thermistor with a resistance of 10 KΩ and an accuracy of 0.5% was installed in the holder of the laser diode for feedback to the temperature controller (TECs), which was 25 mm × 25 mm in size, and pasted on the top and bottom of the laser diode holder. Two heatsinks with a dimension of 40 mm × 40 mm were mounted on the TECs for heat absorption. A control circuit was designed to lock the laser diode’s surface temperature on the reference temperature with very little variation around it.

The real-time recording of the laser diode wavelength with and without stabilization by the proposed RWS were shown in Figure 8. The total sampling time and the sampling frequency were set to be 32 min and 10 Hz, respectively. It can be seen that the stability of laser diode wavelength was improved from 1.2 × 10^−5^ before stabilization to 0.9 × 10^−6^ after stabilization, from which the effectiveness of the proposed RWS was verified. Since the wavelength stability of 10^−6^ is usually required in interferometry measurements [8], the RWS is satisfied for micro/nano positioning stages.

**Figure 9:**
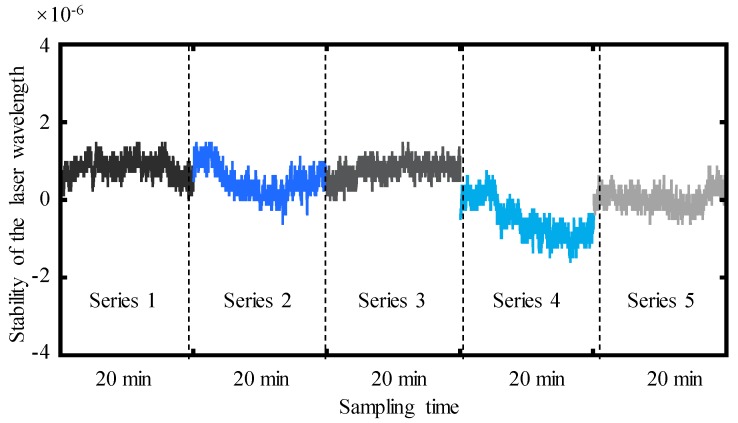
Repeatability of the laser diode wavelength measurements.

Wavelength measurement repeatability is a very important factor for the designed RWC and RWS in the MHI. The measurement repeatability was tested by a group of the wavelength measurement experiments, with which the same laser diode and same parameter of the sampling time and frequency. The sampling time for each experiment series was set to be 20 min. Figure 9 shows the tested results. The repeatability of the laser diode wavelength measurement was at the level of 1.3 × 10^−6^.

### 4.3. Performance of the Miniature Homodyne Interferometer (MHI)

**Figure 10:**
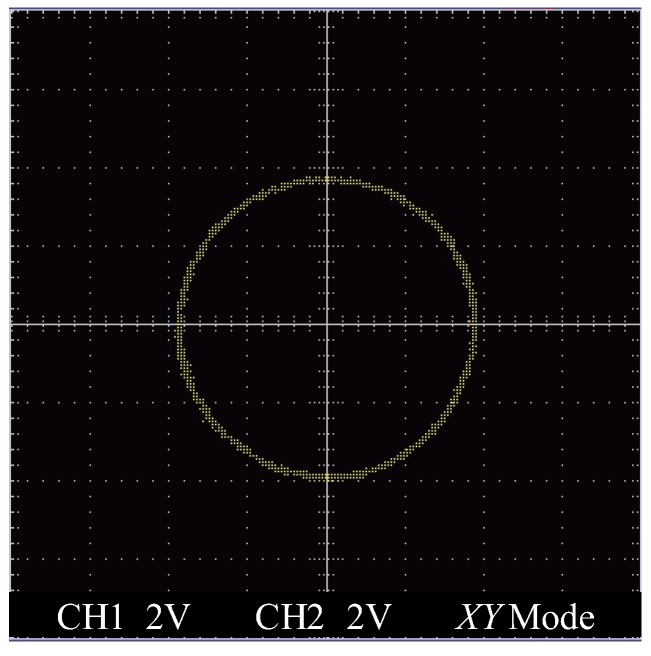
Output Lissajous circle of the MHI.

After investigating the performance of the proposed RWC and RWS, testing of the designed miniature homodyne interferometer (MHI) was carried out. A laboratory-built prototype of the MHI was constructed for long-stroke micro/nano positioning stage metrology. In the MHI, the laser diode was adopted as the laser sources. It has been confirmed that the measurement range of the MHI can reach to 150 mm, which is much longer than our previous work and many other works [4,5,6,7]. Figure 10 shows the output sinusoidal signals in the form of a Lissajous circle, which was found to be very good and stable during measurement range of 150 mm.

**Figure 11:**
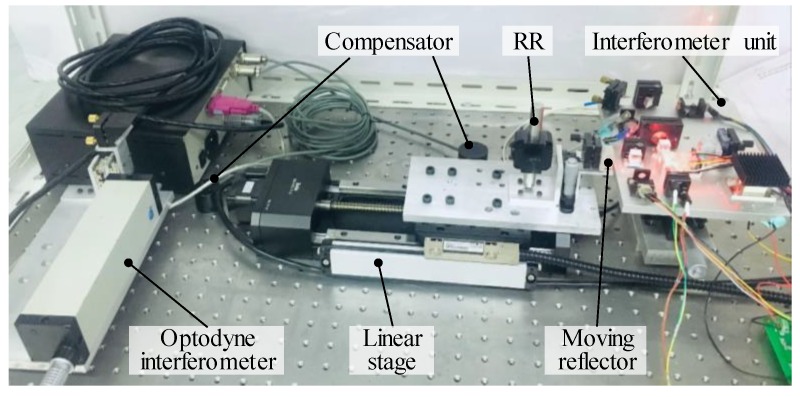
Experimental setup for distance measurement.

As shown in Figure 11, an experimental setup was constructed to verify the feasibility and the measurement accuracy of the MHI. The commercial interferometer (MCV500), with a measurement accuracy of ±0.5 ppm and measurement range of 15 m, was employed as a reference for comparison. The interferometer unit of the MHI and laser head of the MCV500 were positioned on both sides of a precision motorized positioning stage (KA100, Zolix, Beijing, China), which has a moving range of 100 mm, a positioning accuracy of 30 μm, and a repeatability of ±3 μm. The moving reflector of the MHI and the retro-reflector (RR) of the MCV500 were mounted on the moving stage of the positioning stage, which was moved for a distance of 100 mm with a step size of 1 mm at a speed of 2 mm/s. The RWC and RWS were embedded in the MHI and activated during the displacement measurement.

**Figure 12:**
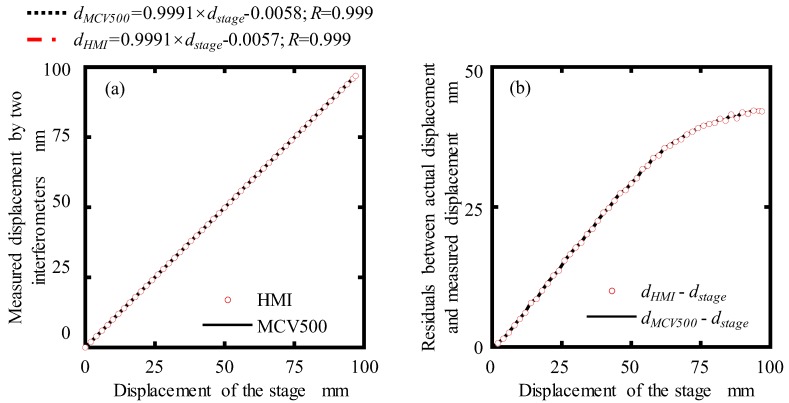
Experiment results for dynamic displacement measurement of a positioning stage: (**a**) measured displacement by the MCV500 and proposed MHI; (**b**) residual between the actual displacement of the stage and the measured displacement by two interferometers.

The measurement axis of the MHI and MCV500 were carefully aligned with the moving axis of the positioning stage so that the Abbe error and cosine error in displacement measurement can be eliminated. The displacement of the positioning stage was simultaneously measured by the MCV500 and the developed MHI. The experiment results for dynamic displacement measurement of a positioning stage are plotted in Figure 12. Figure 12a shows the relationship between the actual displacement (*d_stage_*) of positioning stage and the measured displacements by using the MCV500 (*d_MCV500_*) and proposed MHI (*d_MHI_*). As seen from the figure, the fitted slope and the linear correlation coefficients of two measured displacements are the same. The fluctuating trends of the residuals, which were obtained by making a difference between the actual displacement and measured displacement, are also almost the same, as shown in Figure 12b. This indicates the outputs of the proposed MHI are in a good agreement with those of the commercial laser interferometer.

**Figure 13:**
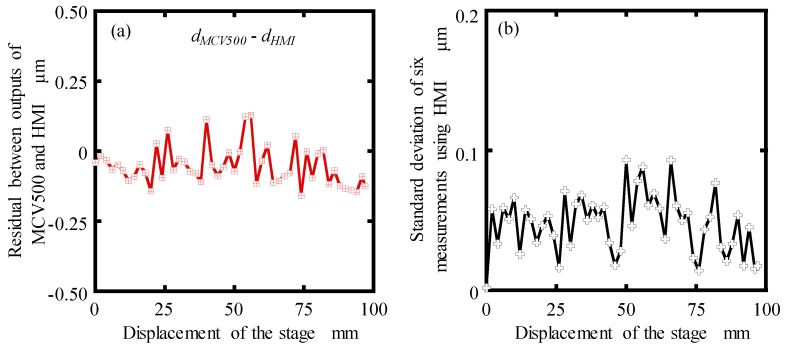
(**a**) Residual between the MCV500 and proposed MHI; (**b**) standard deviation of six displacement measurements by MHI.

In order to get the measurement accuracy of the proposed MHI, the residuals between the measured displacements with the MCV500 (*d_MCV500_*) and proposed MHI (*d_MHI_*) was calculated and shown in Figure 13a. It can be seen that the maximum residual was evaluated within ±130 nm in a measurement range of 100 mm, which is satisfactory for our designed long-stroke micro/nano positioning stage. The standard deviation of the displacement for six measurements using the proposed MHI was estimated between 0.01 μm and 0.09 μm, as shown in Figure 13b. The feasibility of the proposed miniature homodyne interferometer was demonstrated from the experiment results. Therefore, it has been verified that the proposed MHI can be applied to dynamic displacement measurement longer than 100 mm range with a sub-micrometer measurement accuracy.

## 5. Conclusions

This paper presents an innovative low-cost miniature homodyne interferometer (MHI), which is composed of an interferometer unit and a moving reflector. It possesses functions of a laser diode interferometer (LDI) for displacement measurement, a real-time wavelength corrector (RWC) for self-correcting the laser diode wavelength, and a real-time wavelength stabilizer (RWS) for self-stabilizing the laser diode wavelength. A prototype of the MHI was constructed, in which a low-cost and small-sized laser diode was used as the laser source. A series of experiments were carried out. The accuracy of laser wavelength measurement by using the modified wavelength correction equation was achieved to ±0.007 nm and it has been verified that the wavelength measurement accuracy of the wavelength corrector proposed in this research is much higher than that proposed in our previous research. The stability of laser diode wavelength was improved from 1.2 × 10^−5^ before stabilization to 0.9 × 10^−6^ after stabilization by using the proposed RWS. It has been confirmed that the displacement measurement accuracy of the proposed MHI was ±130 nm in a measurement range up to 100 mm.

Although the proposed MHI can be applied to dynamic displacement measurement longer than 100 mm range, the measurement accuracy of the proposed MHI is still lower than that of the commercial laser interferometers. The motion errors of the moving reflector and the linearity error of the interferometer are primary error sources that influence the measurement accuracy of the proposed MHI. The analysis of above-mentioned errors will be carried out in the future works. In addition, the mode hoping locations tend to change over a limited time, which will influence the stability and accuracy of the proposed MHI. This problem will also be considered in the future work.
